# Suggestion of reduced cancer risks following cardiac x-ray exposures is unconvincing

**DOI:** 10.1007/s10654-018-0383-y

**Published:** 2018-03-31

**Authors:** Richard W. Harbron, Claire-Louise Chapple, John J. O’Sullivan, Choonsik Lee, Kieran McHugh, Manuel Higueras, Mark S. Pearce

**Affiliations:** 1 Institute of Health and Society, Sir James Spence Institute, Royal Victoria Infirmary, Newcastle University, Newcastle upon Tyne, NE1 4LP UK; 2 NIHR Health Protection Research Unit in Chemical and Radiation Threats and Hazards, Newcastle University, Newcastle upon Tyne, NE2 4AA UK; 3 Regional Medical Physics Department, Freeman Hospital, Newcastle-upon-Tyne Hospitals NHS Trust, Newcastle upon Tyne, NE7 7DN UK; 4 Paediatric Cardiology, Freeman Hospital, Newcastle upon Tyne Hospitals NHS Foundation Trust, Newcastle upon Tyne, NE7 7DN UK; 5 Division of Cancer Epidemiology and Genetics, National Cancer Institute, National Institutes of Health, Bethesda, MD USA; 6 Radiology Department, Great Ormond Street Hospital for Children NHS Trust, London, UK; 7 Basque Center for Applied Mathematics, Alameda de Mazarredo, 14, 48009 Bilbao, Basque Country Spain

Mohan Doss has provided us with an interesting interpretation of our study investigating cancer risks among young people undergoing cardiac catheterization procedures [1, 2]. As in previous correspondence [3], Doss adjusts expected cancer incidence figures to demonstrate apparently reduced standardised incidence ratios (SIR) for populations exposed to radiation.

We have a number of concerns with this approach. Firstly, Doss has based calculations on the figures presented in Table 3, in which no exclusion period was applied, *i.e.* observed/expected cases accrue immediately after the date of the first recorded procedure. When assessing the impact of radiation exposure, only follow-up after an exclusion period of 5 years (2 for leukaemia/lymphoma) is appropriate.

Secondly, the fact that cancer incidence among young people with congenital heart disease (CHD) is raised compared to the general population is well known and was part of the justification for the study. As we stated in the introduction to our paper, increased cancer incidence may be due to a number of factors, including genetic predisposition, immunosuppression and radiation exposure. No study has ever managed to isolate the relative contribution of these separate risk factors. There are, therefore, no data available from which to determine true ‘background’ rates among individuals with CHD. This includes the study by Lee et al. [4] from which Doss obtains the factor of 1.45 used to adjust expected cancer incidence figures. In particular, Lee et al. do not exclude patients with radiation exposure nor censor transplant recipients. Furthermore, given the variation in both CHD rates [5] and cancer incidence [6] between countries, the use of data from Taiwan to adjust background figures representing the UK, Canada and Israel is likely unreliable. It is also unclear why Doss has picked the SIR reported by Lee et al., as opposed to other, more modestly raised SIR figures representing populations with CHD (*e.g.* those reported by Bjørge et al. [7]).

Our conclusion that radiation exposure may still contribute to higher cancer rates among children with CHD was partly based on an internal dose response analysis, in addition to calculation of SIR. While imprecise, the excess elevated risk (ERR) per mGy for lympho-haematological neoplasia of 0.018 (95% CI: -0.002, 0.096) is suggestive of a small risk due to radiation exposure. Furthermore, we must again emphasise that follow-up times were insufficient to allow conclusions to be drawn on cancer incidence for the most heavily irradiated organs, including the lungs and breasts.

While Doss is correct in stating that leukaemia incidence is higher among individuals with Down syndrome, the incompleteness of information on prevalence of this condition in our cohort prevented us from formal analysis of the potential for confounding. Doss states that “all the four leukemia cases [….] were in patients with Down’s syndrome”. This is incorrect, although our phrasing could have been clearer. We merely stated that all four cancer cases developing among individuals with Down syndrome were leukaemia. In fact only one of these diseases developed more than 2 years following the first procedure, thus contributing to dose response analysis.

In summary, while we appreciate Dr Doss’s interest in our study, we feel that the methods used to adjust our SIR figures are inappropriate and we are unconvinced of the implied suggestion that radiation exposure in this patient group may be reducing cancer risks via a hormesis mechanism.
